# Reference genes to study the sex-biased expression of genes regulating *Drosophila* metabolism

**DOI:** 10.1038/s41598-024-58863-5

**Published:** 2024-04-25

**Authors:** Sofie De Groef, Melanie Ribeiro Lopes, Mattias Winant, Emily Rosschaert, Tom Wilms, Lenz Bolckmans, Federica Calevro, Patrick Callaerts

**Affiliations:** 1 KU Leuven, Department of Human Genetics, Laboratory of Behavioral and Developmental Genetics, Campus Gasthuisberg O&N1, Herestraat 49 Box 610, 3000 Leuven, Belgium; 2 INSA Lyon, INRAE, BF2i, UMR0203, F-69621 Villeurbanne, France

**Keywords:** Reverse transcription polymerase chain reaction, Animal physiology

## Abstract

Sex is an important variable in biology. Notable differences have been observed between male and female *Drosophila* in regulation of metabolism, in response to nutritional challenges, and in phenotypes relevant for obesity and metabolic disorders. The differences between males and females can be expected to result from differences in gene expression. We observed that expression levels of reference genes commonly used for normalization of qRT-PCR results such as GAPDH, β-actin, and 18SrRNA, show prominent sexual dimorphism. Since this will impact relative expression and conclusions related to that, we performed a systematic analysis of candidate reference genes with the objective of identifying reference genes with stable expression in male and female *Drosophila*. These reference genes (*LamCa*, *βTub60D* and *βTub97EF*) were then used to assess sex-specific differences in expression of metabolism associated genes. Additionally, we evaluated the utility of these reference genes following a nutritional challenge and showed that *LamCa* and *βtub97EF* are stably expressed between sexes and under different nutritional conditions and are thus suitable as reference genes. Our results highlight the importance of evaluating the stability of reference genes when sex-specific differences in gene expression are studied, and identify structural genes as a category worth exploring as reference genes in other species. Finally, we also uncovered hitherto unknown sexually dimorphic expression of a number of metabolism-associated genes, information of interest to others working in the field of metabolic disorders.

## Introduction

It is increasingly recognized that sex underlies important differences in animal biology, physiology, and pathology. Nevertheless, in most studies, results obtained in only one sex are extrapolated to both sexes, thereby unintentionally neglecting possibly relevant and important sex-specific differences in the underlying mechanisms^1^. Morphological, behavioral and physiological differences between males and females in somatic and gonadal tissues are primarily the result of differences in gene expression of autosomal genes, orchestrated by hormones and sex chromosomes^2^. Sex-specific expression is not a fixed feature of a given gene but is highly tissue-dependent and variable over the course of development and under specific conditions^3,4^. Additionally, detection and classification of sex-biased genes is also dependent on technical aspects, such as the method used to measure gene expression, the quality and number of samples, the methods used for data processing and analysis, and the statistical approach used^5^.

Quantitative reverse transcription-polymerase chain reaction (qRT-PCR) is an efficient method to study gene expression by measuring absolute or relative mRNA levels in a wide range of biological samples. To assess relative gene expression, it is crucial to perform accurate normalization against so-called “reference genes” or “housekeeping genes”, that are involved in basic cellular functions. These genes are expected to be transcribed in a stable fashion across different cell types and organs, and not be affected by conditions such as age, sex, or experimental treatments^4,6–8^. Finding a good normalization gene is nonetheless difficult, and many genes classically used in mRNA level normalization have since been shown to vary in specific tissues, cells, and stress or disease conditions^9–11^. Hence, today, reference genes need to be experimentally validated for their stability in the tested organism, conditions, and samples. Several statistical algorithms have been developed to determine the stability of reference genes, including the Delta Ct comparative method^12^, geNorm^13^, BestKeeper^14^, NormFinder^15^ and RefFinder^16,17^. The assumption of these statistical algorithms is that there is no systematic variation in expression of the reference genes. However, the sex of individuals used to produce the sample can be an important source of variation, including for the expression of reference genes^18^.

*Drosophila melanogaster* is used extensively as a genetically tractable model organism for the study of metabolism^19^, lifespan^20^, cancer^21^, and immune response^22^. Notable differences have been observed between female and male *Drosophila* in regulation of metabolism, in responses to nutritional challenges and in the occurrence of phenotypes relevant for obesity and metabolic disorder^23–29^. Those differences can be expected to result from differences in gene expression. In our previous study on the sexually dimorphic effects of Western diet, we noticed that frequently used reference genes for qRT-PCR are themselves sexually dimorphic^29^. The use of these genes for normalization will introduce a bias and can exaggerate or diminish actual differences in gene expression thus leading to incorrect conclusions. To remedy this, we set out to identify stably expressed references genes to study the sex-biased expression of genes regulating *Drosophila* metabolism. To do that, we analyzed and tested nine candidate genes by means of reference gene stability calculators in order to identify those displaying stable expression in male and female *Drosophila* head and body (thorax + abdomen), in two wildtype strains (Canton S-10 and Dahomey) at 2 and 7 days post-hatching. The most stable reference genes were *LamCa*, *βTub60D* and *βTub97EF* while other commonly used reference genes like those encoding ribosomal proteins (*RpL32* and *RpS13*) revealed significant sex-bias. We used *LamCa*, *βTub60D* and *βTub97EF* to assess the sex-specific differences in expression of metabolism-associated genes. Additionally, we tested the utility of these reference genes following a nutritional challenge and found that two (*LamCa* and *βTub97EF*) were appropriate for studying sex-specific responses after starvation. Our data highlight the importance of evaluating the stability of reference genes in different experimental contexts, especially when sex-specific differences in gene expression are studied. We also uncovered sexually dimorphic expression of 10 metabolism-associated genes and identified genes encoding structural components of the cell and nucleus as good candidates to be explored as reference genes in other species.

## Results

### Commonly used reference genes display sex-biased expression

**Figure 1:**
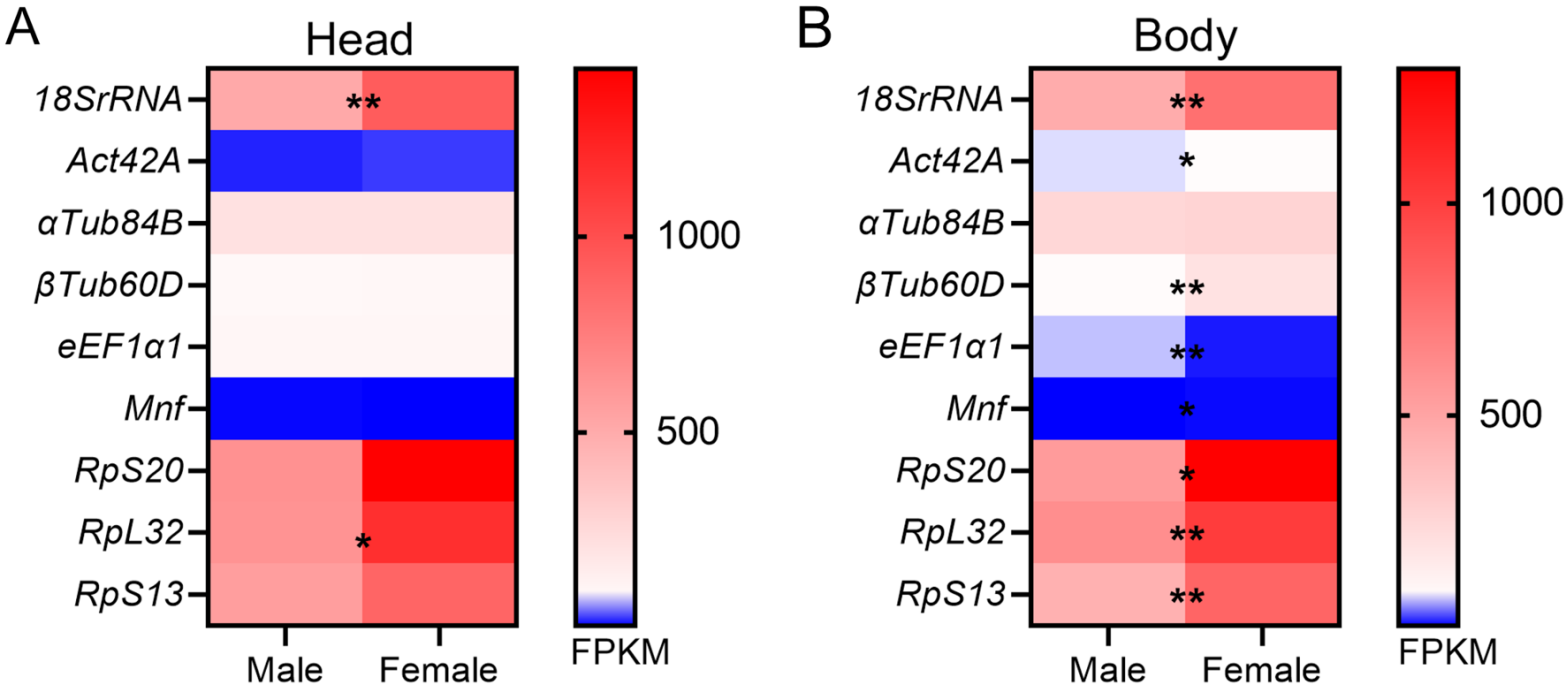
Heatmap depicting FPKM (Fragments Per Kilobase Million) data in adult *Drosophila* male and female for commonly used reference genes in head (**A**) and whole body (**B**). FPKM data and statistics obtained from the FlyAtlas2 database.

We used the FlyAtlas2 database^43^ to assess expression data relative to commonly used *Drosophila melanogaster* reference genes *18SrRNA, Actin42A, α Tubulin 84B (αTub84B), β Tubulin56D (βTub56D), eukaryotic translation elongation factor 1 alpha 1 (eEF1α1), Myocyte nuclear factor (Mnf* = *FoxK/Forkhead box K), RpS20, RpL32* and *RpS13*^4,30^. We observed that most reference genes display a sex-biased expression. This sex-bias is most prominent and significant for genes encoding ribosomal subunits in whole body samples (Fig. 1A,B). *α Tub84B* is the only gene that does not display sexual dimorphism in whole body (Fig. 1B). We hypothesized that other genes encoding for “structural components of the cell or nucleus” could be potentially interesting candidates for reference genes without sex bias. We used FlyBase^31^ to identify the genes belonging to this category. This yielded 137 genes of which 11 are expressed in the adult stage (Table 1). We assessed whether these 11 genes had ubiquitous expression and/or showed whole-body sex bias. For two of these genes, *CG32820* and *CG32819*, no expression data were available. Based on ubiquitous expression in all tissues and no or limited sexually dimorphic expression, we selected *LaminCa*, *Actin-related protein 3* (*Arp3*) and *β Tubulin 97EF (β Tub97EF)* as potential additional candidate reference genes without sex-bias in gene expression for testing and comparison to *Actin42A*, *αTub84B, βTub60D, eEF1α1, RpL32* and *RpS13*.

**Table 1:** FPKM from FlyAtlas database for candidate genes in the “structural constituent of cytoskeleton” group.

Flybase ID	Gene	Name	GO_Biological process	Whole body male (FPKM)	Whole body female (FPKM)	Stat. significant difference male–female	Expression
FBgn0010097	γ-Tubulin at 37C	γTub37C	Cytoplasmic microtubule organisation	0	24	p < 0.01	Enriched in ovary, female salivary gland and heart
FBgn0010397	LaminCa	LamCa	Muscle tissue morphogenesis	9.9	7.2	p < 0.05	Ubiquitous
FBgn0003886	α-Tubulin at 85E	αTub85E	Unidimensional cell growth	6.4	0.4	p < 0.01	Enriched in testis
FBgn0262716	Actin-related protein 3	Arp3	Actin filament reorganization involved in cell cycle	14	22	p < 0.05	Ubiquitous
FBgn0051363	Jupiter	Jupiter	Positive regulation of microtubule polymerization cell morphogenesis	51	35	ns	Enriched in testis
FBgn0011742	Actin-related protein 2	Arp2		15	34	p < 0.01	Ubiquitous
FBgn0003890	β-Tubulin at 97EF	βTub97EF	Mitotic cell cycle	24	13	ns	Ubiquitous
FBgn0052820	CG32820	–	Microtubule nucleation microtubule nucleation regulation of actin polymerization regulation of actin polymerization				
FBgn0052819	CG32819	–					
FBgn0032859	Actin-related protein 2/3 complex, subunit 2	Arpc2		9.7	39	p < 0.01	Ubiquitous
FBgn0038369	Actin-related protein 2/3 complex, subunit 3A	Arpc3A		8.7	15	p < 0.01	Ubiquitous

### Ct-values for candidate reference genes vary with age, sex and strain

**Figure 2:**
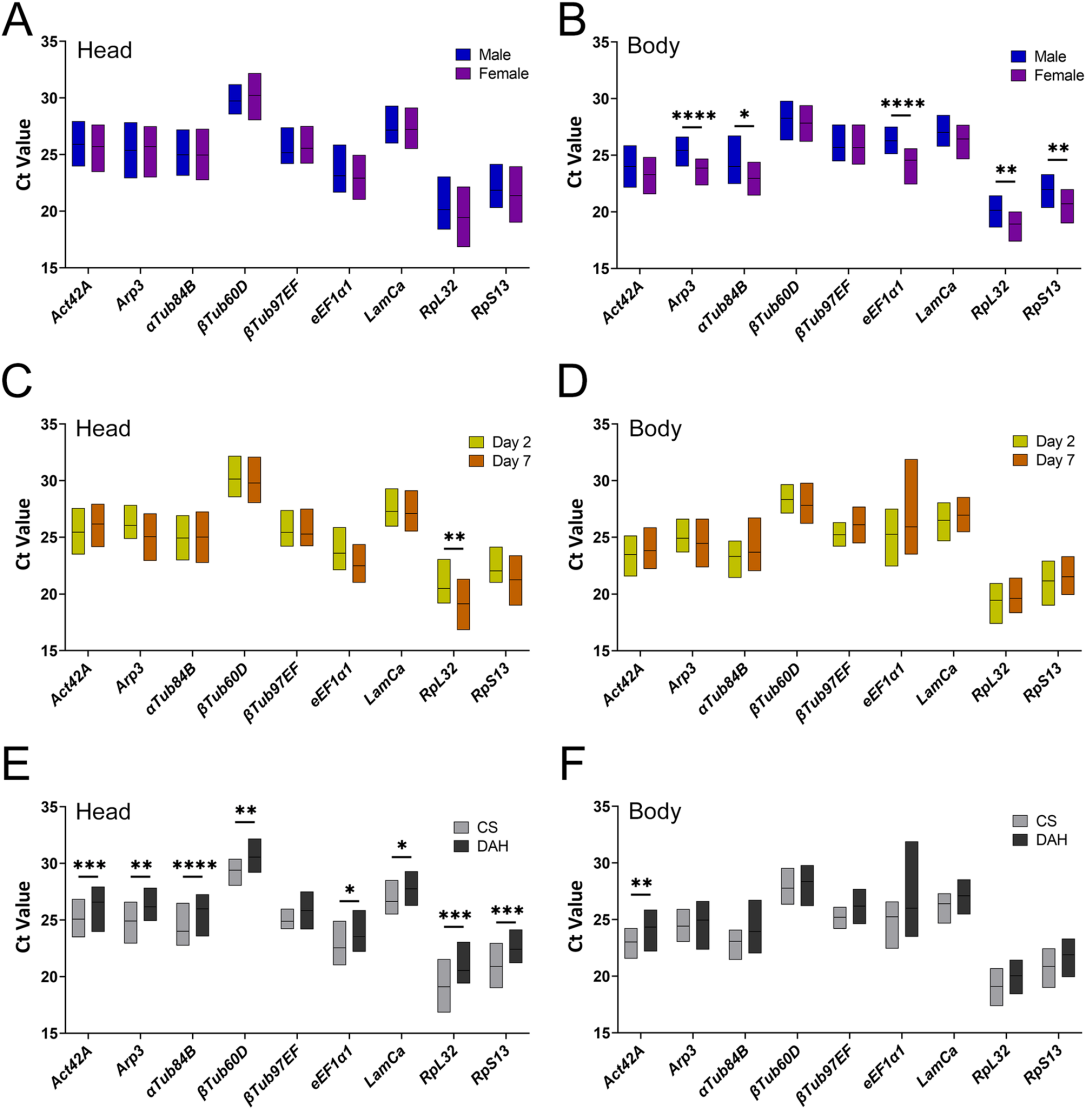
Ct-values for 9 reference genes in the heads (**A**,**C**,**E**) or bodies (**B**,**D**,**F**) of adult *Drosophila* males (**A**) or females (**B**), 2- (**C**) or 7-days post eclosion (**D**), in CantonS 10 (**E**) or Dahomey strains (**F**). *p < 0.05, **p < 0.005, ****p < 0.0001.

The expression levels of the reference genes were detected as cycle threshold (Ct) values. First, we validated the primer efficiency (Table 2) and expression of *Actin42A*, *Arp3*, *αTub84B, βTub60D, βTub97EF, eEF1α1, LaminCa, RpL32* and *RpS13*. We performed qRT-PCR on male and female head and body (thorax + abdomen) from CS10 and Dahomey flies collected 2 and 7 days after hatching. Table 3 displays the mean Ct-values, the standard deviation, and the coefficient of variation for each gene across all conditions. The mean Ct values over all conditions ranged from 19.546 (*RpL32*, body) and 29.964 (*βTub60D*, body). Standard deviation ranged from 0.873 (*LaminCa*, body) to 1.651 (*eEF1α1,* body). In both head and body samples *βTub60D, βTub97EF* and *LaminCa* displayed the lowest standard deviation and coefficient of variation. Next, we evaluated statistical differences in Ct-values for each gene between males and females (Fig. 2A,B), between day 2 and day 7 post eclosion (Fig. 2C,D) and between CS10 and Dahomey strains (Fig. 2E,F). Two-way ANOVA revealed that sex, age, and strain statistically contribute to the variation in the data (see Supplemental Data for results of two-way ANOVA). Ct-values for genes in head tissue did not show statistically significant differences between males and females, while in body *Arp3*, *αTub84B*, *eEF1α1*, *RpL32* and *RpS13* expression displayed a female bias*.* Remarkably, Ct-values for genes in head displayed statistically significant differences between CS10 and Dahomey flies, with higher Ct-values for all genes in Dahomey flies (Fig. 2E).

**Table 2:** Primer sequences and primer efficiency.

Gene name	Forward primer (5'-3')	Reverse primer (5'-3')	Efficiency head	Efficiency body	Efficiency whole fly
Act42A	TGCAAAAGGAAATCACGGCG	CCGCCGATCCAAACAGAGTA	97	105	
Arp3	ACTACTTCCTGCTGACTGAG	CCTGGACGGCGATATACA	132	93	
aTub84B	GGAGTTCGCCATCTACCCAG	ACTGATTTCGACGGTTACCCC	83	78	
bTub60D	CAAATCGGCGCTAAGTTCTGG	CCCACGTAGATGCCATTGCT	91	109	
bTub97EF	CCGCATCATGAACTCCTTCT	TGGACAGCGTGGCATTGTAC	119	94	
eEF1a1	AGGCCGCCTAAATTGGGAAA	ATCGGAGGCAACAAGCAAA	98	152	
LaminCa	TCCACCCAACAATCTGGTGAT	GCAACATCCTCTTTGTCGGC	94	100	
RpL32	GTCCCAAGGGTATCGACAACA	CTTGCGCTTCTTGGAGGAGA	95	102	
RpS13	GGGTCTGAAGCCCGACATT	GGCGACGGCCTTCTTGAT	93	96	
dIlp2	AGCAAGCCTTTGTCCTTCATCTC	ACACCATACTCAGCACCTCGTTG	93		
dIlp3	GCAATGACCAAGAGAACTTTGGA	GCAGGGAACGGTCTTCGA	93		
dIlp5	GCTCCGAATCTCACCACATGAA	GGAAAAGGAACACGATTTGCG	94		
Bmm	AATGGCGTCGAATCAGACTT	AACACAGATGGGGATTTGGA		126	
dIlp6	TGCTAGTCCTGGCCACCTTGTTCG	GCTTCCCGAAACTGTTGGGAAATA		128	
Fit	TCGTTGGTCTGAGGAGGACA	CCAGTTGACAGAGTGCGGAT		105	
Foxo	AAATTCGTCTATCGGCTGCG	TTGGAAGATAATAACTGCGCCTCT		106	
Drs	ACCAAGCTCCGTGAGAACCTT	TTGTATCTTCCGGACAGGCAG		109	
Lpin	ATCCCACGTCCCTGATATCG	TTCATCTTGGTTGGTTAGCAGG			104
FASN1	GTTGGGAGCGTGGTCTGTAT	GCACACCGAAGAACTGTTGG			97
ACc	GGCTATGCTGCGCTTAACA	GCCTCTGTTTTGTGGGTGAC			85
HSL	GATCCATTCCTGTCGCCGTA	CAGGGGTCCATGTTAAGTGTAAGT			102
LSD1	TCACAATCTCACGGCTGGAC	GGCTACCATAGAACGCCAGC			112
LSD2	CGGATGTCGAGGAAGATAATGATGA	GTCGTAGCTCTCCCCAACAA			128
ATPCL	CTTCTGACCATCGGGGATCG	CAGGTTGGTGTCGTATGCCT			95
Sea	ACATGAAGGAGCTGGGCGTC	CCAGGGTCTCCTCGTCGAAC			93
Pgi	GCATTCCAAGGAGCCTGAGT	ATGTTGGTGCTGTCAATGCC			111
Tpi	CATCAAGGACCTGTTCGTGAA	AGTCCAGCAGTATCTCGCCAT			103
PEPCK	GCCTGAGCTCATTGAACAAAG	ACCGCAATTGTCCTGGCGCA			101
bigmax	GCCAAGTTTCAAGTGTTCCAG	CTCCAGCCAGGGGATAATG			112
GADPH2	ATGAAATTAAGGCCAAGGTT	GAGTAGCCAAACTCGTTGTC			102
GADPH1	CTCGACTCACGGTCGTTTCA	GGTGATCTTCTGGCCGTTCA			59
Upd2	AGTGCGGTGAAGCTAAAGACTTG	GCCCGTCCCAGATATGAGAA			83
zw	ATCTGTGCGGGAAGTGATG	CCAAACTTGGCTTCGGAAC			84
Pgd	ATGAGCGGACAAGCGGATATT	TAGGCGCACACCACGAATC			98
GlyS	AGTCCTACTTCATCGCGGCA	GTCTCCTCATCCACGGAGTC			81
GlyP	ATACAACAACAACCACGTAAACAC	GGATGTAGTCACCATCGTTGAAG			97
Tps1	TTCCTGGACATCCCATTCCC	TCACAACCCAACATACCCTGT			105
Eiger	TCCTAGTCCGCAAAGGTGAA	CAAGTGGAAATGGGCTGCTG			82
TACE	AGAATTGCGCGGCCCTG	CTTGGCACCCCTTTTCACCA			103
cchamide	CGCCAAATGAACAGGTGCC	GTCGGCGAGGTCGGTTAAA			104
Pgk	TCATCGTGTGGAACGGACCC	AGACGAGTGCCTCCGTGTTC			90
Rel	TTCCGTGAAAAGCTGACCCG	GAGTGGGCTGGTGAAAAGGA			104
STAT92E	CAGGGCGTTCTCTGACCATT	TTCGGGCTCTTGACGCTTTG			100

**Table 3:** Mean Ct-values, standard deviation, and coefficient of variation for each reference gene across all conditions.

	HEAD			BODY		
Gene	Mean	SD	Coeff of var	Mean	SD	Coeff of var
*Act42A*	25.811	1.227	0.048	23.658	1.079	0.046
*Arp3*	25.532	1.153	0.045	24.682	1.108	0.045
α*Tub84B*	24.969	1.458	0.058	23.503	1.079	0.046
β*Tub60D*	29.964	0.969	0.032	28.062	1.007	0.036
β*Tub97EF*	25.349	0.882	0.035	25.682	0.962	0.037
*eEF1a1*	23.022	1.147	0.050	25.611	1.651	0.064
*LamCa*	27.187	1.025	0.038	26.738	0.873	0.033
*LamCb*	30.582	1.106	0.036	30.327	0.850	0.028
*RpL32*	19.806	1.383	0.070	19.546	1.025	0.052
*RpS13*	21.630	1.241	0.057	21.359	1.110	0.052

### Stability of gene expression

Next, we evaluated the stability of the reference genes in head and body samples across all conditions using statistical algorithms: comparative Delta Ct method, Normfinder, Bestkeeper and Genorm. For each analysis, except for NormFinder, all samples were used irrespective of sex, age, and strain. The calculation by NormFinder required subgroup specification. Therefore, age, sex and strain were set as subgroups for the analysis, leading to 8 subgroups.

**Figure 3:**
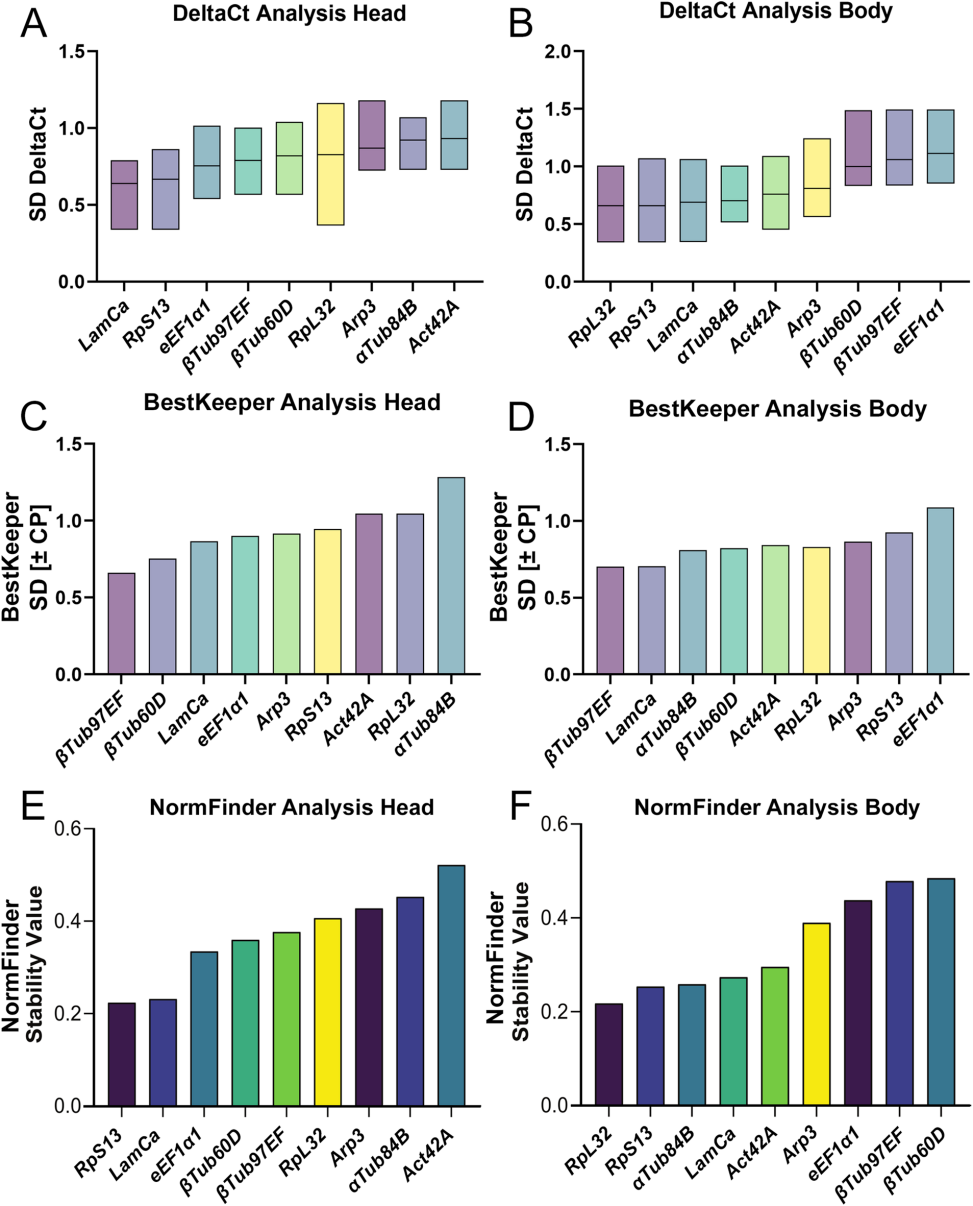
(**A**,**B**) Delta Ct method values for head (**A**) and body (**B**) samples. Bars depict mean standard deviation of the differences in the paired comparisons of each gene in each sample (level of deviation). Ranking the reference genes from more stable (left) to less stable (right). (**C**,**D**) Bestkeeper method values for head (**C**) and body (**D**) samples. Bars depict Bestkeeper- stability value ranked according the crossing point standard deviation value (Std dec [+− CP]. Ranking the reference genes from more stable (left) to less stable (right). (**E**,**F**) Normfinder values for head (**E**) and body (**F**) samples. Bars depict Normfinder- stability value ranking reference genes according to the lowest intra- and intergroup variation. Ranking the reference genes from more stable (left) to less stable (right).

The Delta Ct comparative method compares relative expression of “pairs of candidate genes” within each sample. If the Delta Ct-value (difference between two genes) remains constant when analyzed in different samples, this means that the genes are stably expressed. If the Delta Ct fluctuates across samples, one or both genes are variably expressed. The standard deviation of Delta Ct values can be calculated for each gene across the samples. The mean of the standard deviation provides a value that describes the variability, with lower values corresponding to more stable expression. Figure 3A and B display standard deviation of Delta Ct analysis for candidate references genes in head and body samples, across all conditions (sex, age, strain). In head tissue, all values were below 1, with genes *LaminCa* (0.63), *RpS13* (0.67) and *eEF1α1* (0.75) displaying the lowest variability. In body samples lowest values were observed for *RpL32* (0.66), *RpS13* (0.66) and *LaminCa* (0.69). The Delta Ct method compares Ct-values between two genes within one sample, in case that a comparable sex-bias, age bias, or strain bias is observed for multiple reference genes, the delta Ct value will not fluctuate with sex, age or strain.

Bestkeeper software calculates an index using the geometric mean of raw Ct*-*values for each candidate gene. Gene expression variation can be determined by the calculated standard deviation (*SD*) and coefficient of variance (*CV*) for all candidate reference genes based on their Ct-values. Candidate genes with *SD* values greater than 1 were considered as inconsistent and were excluded. Then the Bestkeeper program estimated the relationship between the index and the contributing reference gene by the Pearson correlation coefficient, the coefficient of determination (*r*^2^), and the *P* value. The larger r, the smaller the SD and CV, the better the stability of the reference gene. Bestkeeper analysis displays SD for head (Fig. 3C) and body samples (Fig. 3D), suggesting *βTub97EF*, *βTub60D*, *LaminCa* as most stable reference genes in head and *βTub97EF*, *LaminCa* and *αTub84B* in body samples.

NormFinder determines the stability of the candidate reference genes by measuring the intra- and intergroup variation between specified groups. Here, we labeled every sex, strain, and age as a separate group, leading to 8 groups in total. Stability values for each candidate gene are calculated by adding the two sources of variation. The lowest stability value indicates the most stable expression. For head samples, the lowest stability value was *RpS13* (0.224), *LaminCa* (0.232) and *eEF1α1* (0.0335), the best combination of two genes is *LaminCa* and *RpS13* with stability value of 0.178. (Fig. 3E). For body samples the lowest stability value was *RpL32* (0.218*), RpS13* (0.254) and *αTub84B* (0.259), the best combination of two genes is *LaminCa* and *RpL32* with stability value of 0.162. (Fig. 3F).

**Figure 4:**
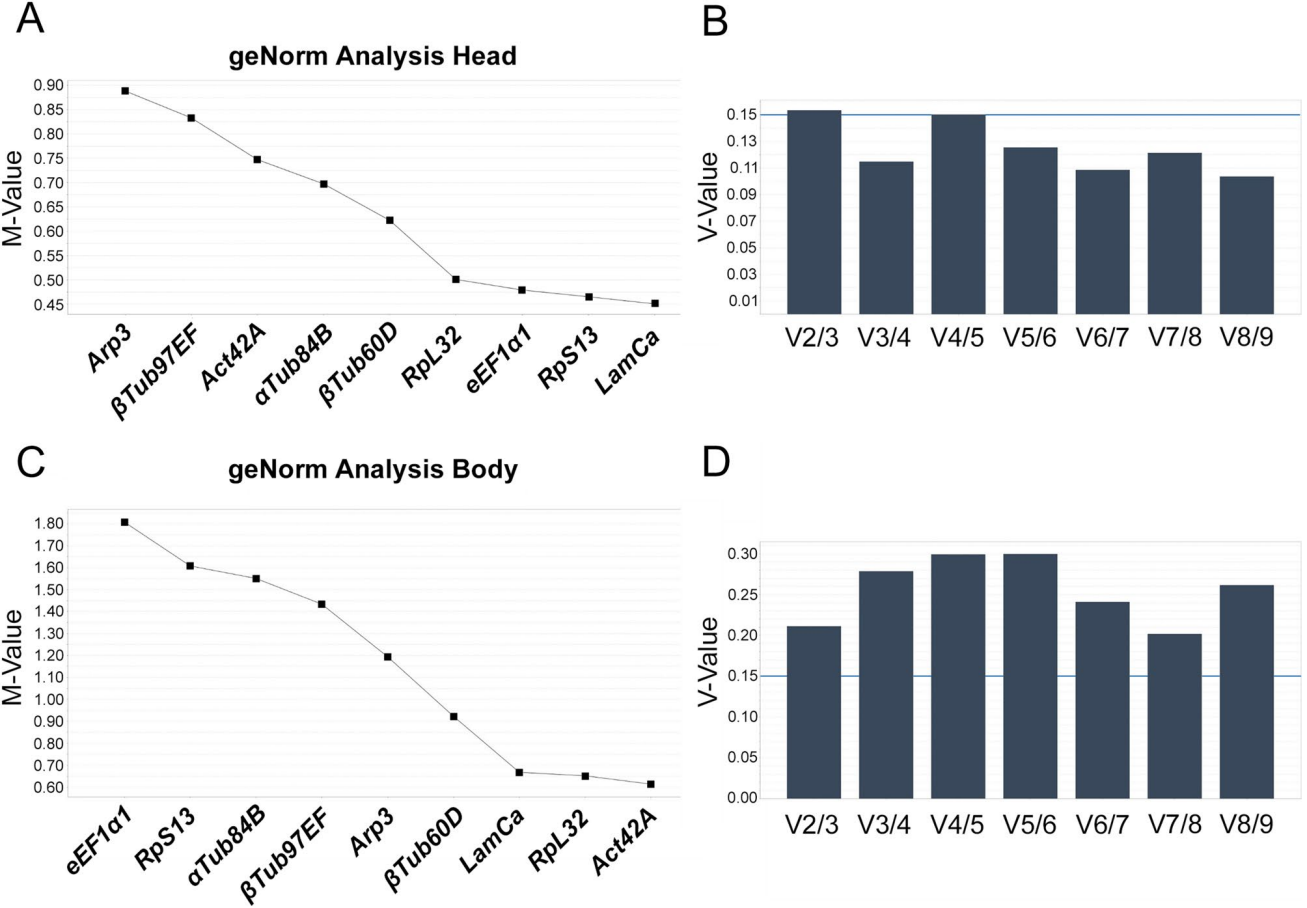
Output of the geNorm algorithm for head (**A**,**B**) and body (**C**,**D**) samples. Ranking of the reference genes according to their M-value for head (**A**) and body (**C**) samples, with references genes with the lowest M value assumed to be the most stable. geNorm pairwise variation (V) analysis of 9 reference genes to determine optimal number of reference genes for normalization of head (**B**) and body (**D**) samples.

geNorm calculates expression stability value (M value) for a candidate reference gene based on the geometric mean of all studied genes in a pairwise comparison. The reference gene with the lowest M value should be the most stable gene and an M value under 1.5 is suggested by the geNorm software as a criterion for the selection of the reference gene(s). While a gene can display low pair-wise variation, geNorm software does not allow defining groups and calculate the intergroup variation. geNorm also allows to calculate the optimal number of reference genes by determining the pairwise variation between the sequentially ranked genes (Vn/Vn + 1) based on the geNorm algorithm. A cut-off of 0.15 (Vn value) is recommended, below which the inclusion of additional reference genes is not required. Thus, if V_n/n+1_ < 0.15, it is not necessary to use ≥ n + 1 reference genes as internal controls. Figure 4A and B display the M-values and Vn-values for head samples, respectively. The optimal number of reference genes in head samples is 3 (Vn -value 0.115), with *LaminCa, RpS13* and *eEF1α1* as reference genes with lowest M-value. *Act42A, RpL32* and *LaminCa* display the lowest M-values in body samples (Fig. 4C). For these samples, all Vn-values were higher than the cut off 0.15, suggesting that additional reference genes should be included in the analysis to determine the optimal number of reference genes (Fig. 4D). Using three reference genes for analysis would allow the lowest Vn-value (0.21).

### Optimal reference genes to evaluate sex-specific differences

**Figure 5:**
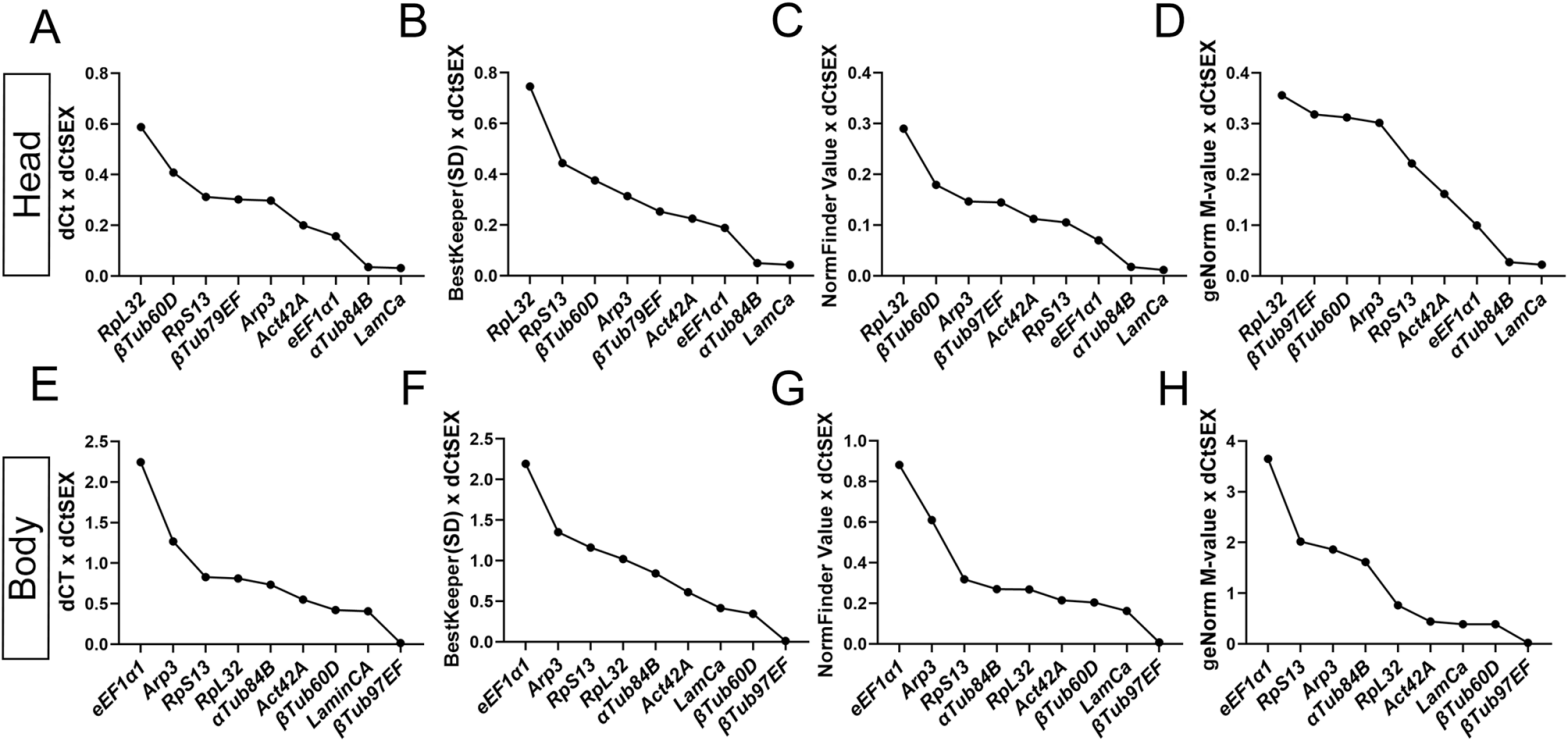
Graphs depicting the calculation of most stable reference genes according to delta-Ct method (**A**,**E**), bestkeeper (**B**,**F**), normfinder (**C**,**G**) and geNorm (**D**,**H**), multiplied with the absolute difference in mean CT value of the reference genes in males versus females (deltaCTSEX) in head (**A**–**D**) and body (**E**–**H**).

To incorporate the sex-specific bias in Ct-values of reference genes, St-Pierre et al. used the geNorm M-value and multiplied it with the absolute difference in mean Ct-value between male and female samples^18^, henceforth named “deltaCtSEX”^18^. For head and body samples we multiplied the value obtained in the delta Ct comparison method (Fig. 5A and E), Bestkeeper value (Fig. 5B and F), stability value from Normfinder (Fig. 5C and G) and the M-value from geNorm (Fig. 5D and H) with the deltaCtSEX. Tables 4 and 5 give an overview of the three most stable reference genes as calculated by each of the algorithms. Multiplication of the obtained values with the deltaCtSEX shows that reference genes *LaminCa*, *αTub84B* and *eEF1a1* and *βTub97EF*, *LaminCa* and *βTub60D* for head and body samples respectively, display the lowest variation and the smallest difference in Ct-values between sexes.

**Table 4:** Summary table displaying the 3 most stable reference genes obtained from all the used algorithms and calculations in head samples.

HEAD	1	2	3
DeltaCt	LaminCa	RpS13	eEF1a1
BestKeeper	βTub97EF	βTub60D	LaminCa
NormFinder	RpS13	LaminCa	eEF1a1
geNorm	LaminCa	RpS13	eEF1a1
deltaCt*dCtSEX	LaminCa	αTub84B	eEF1a1
BestKeeper*dCtSEX	LaminCa	αTub84B	eEF1a1
NormFinder*dCtSEX	LaminCa	αTub84B	eEF1a1
Mvalue*dCtSEX	LaminCa	αTub84B	eEF1a1

**Table 5:** Summary table displaying the 3 most stable reference genes obtained from all the used algorithms and calculations in body samples.

BODY	1	2	3
DeltaCt	RpL32	RpS13	LaminCa
BestKeeper	βTub97EF	LaminCa	αTub84B
NormFinder	RpL32	RpS13	αTub84B
geNorm	Act42A	RpL32	LaminCa
deltaCt*dCtSEX	βTub97EF	LaminCa	βTub60D
BestKeeper*dCtSEX	βTub97EF	βTub60D	LaminCa
NormFinder*dCtSEX	βTub97EF	LaminCa	βTub60D
Mvalue*dCtSEX	βTub97EF	βTub60D	LaminCa

### Sex-biased expression of metabolism genes in head and body samples.

**Figure 6:**
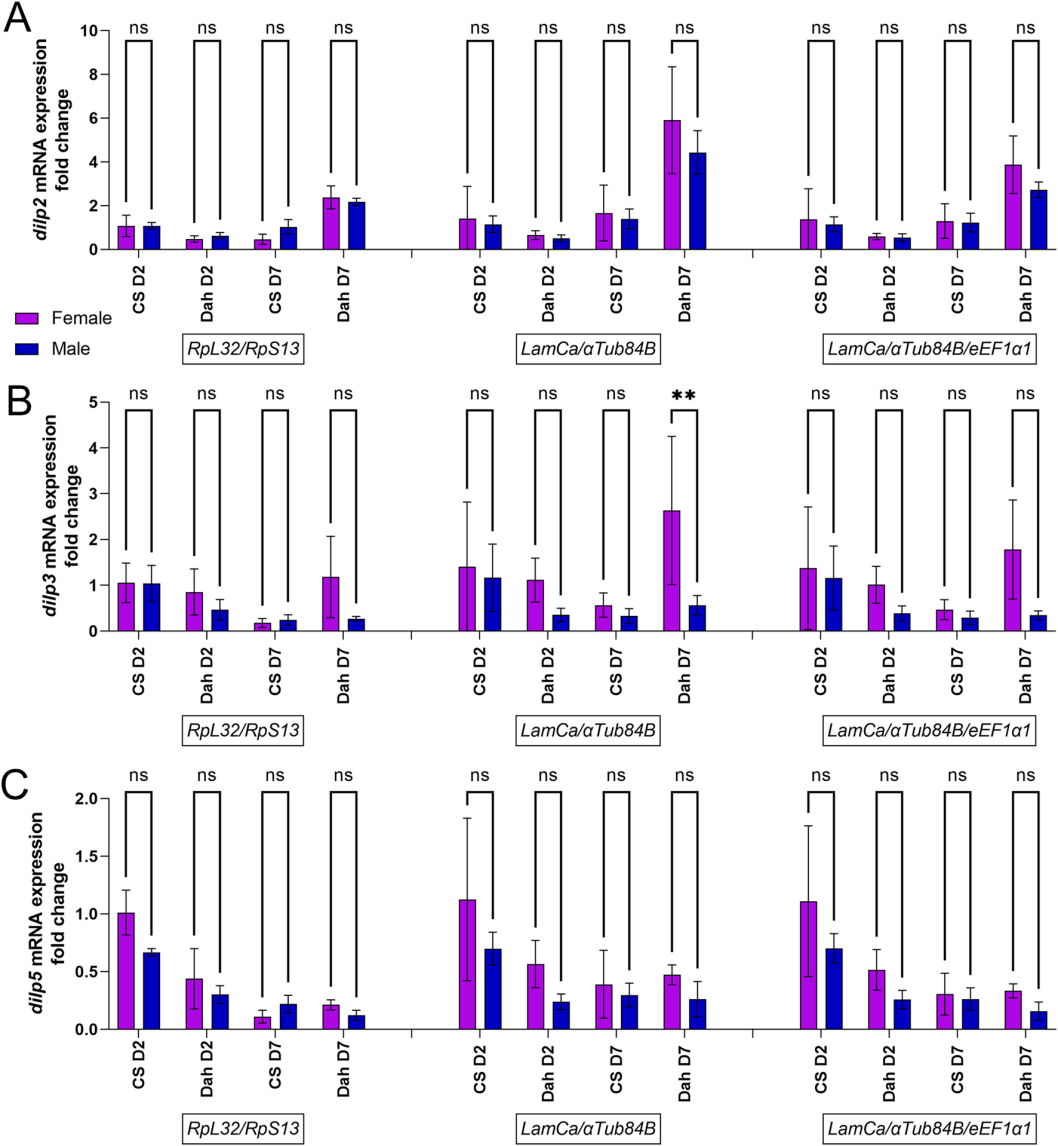
(**A**) Gene expression analysis for *dIlp2* mRNA expression in head samples of Canton S10 (CS) or Dahomey (Dah) flies, 2 days (D2) or 7 days (D7) post eclosion in male and female flies. Gene expression was normalized using ribosomal genes as reference genes (RpL32/RpS13), *LaminCa*/*αTub84B* or *LaminCa/αTub84B/eEF1α1*. Two-way ANOVA was performed with effects as follows: normalization; p < 0.0001; sex; ns and their interaction; ns with a Sidak’s multiple comparisons test comparing *dIlp2* expression between males and females. (**B**) Gene expression analysis for *dIlp3* mRNA expression in head samples of Canton S10 (CS) or Dahomey (Dah) flies, 2 days (D2) or 7 days (D7) post eclosion in male and female flies. Gene expression was normalized using ribosomal genes as reference genes (*RpL32*/*RpS13*), *LaminCa/αTub84B* or LaminCa/*αTub84B*/*eEF1α1*. Two-way ANOVA main effects as follows: normalization; p = 0.0032; sex p < 0.0001 and their interaction; ns with a Sidak’s multiple comparisons test comparing dilp3 expression between males and females. **p < 0.005. (**C**) Gene expression analysis for *dIlp5* mRNA expression in head samples of Canton S10 (CS) or Dahomey (Dah) flies, 2 days (D2) or 7 days (D7) post eclosion in male and female flies. Gene expression was normalized using ribosomal genes as reference genes (*RpL32*/*RpS13*), *LaminCa/αTub84B* or *LaminCa/αTub84B*/*eEF1α1*. Two-way ANOVA main effects as following: normalization; p < 0.0001; sex p < 0.0001 and their interaction; ns with a Sidak’s multiple comparisons test comparing dilp3 expression between males and females.

**Figure 7:**
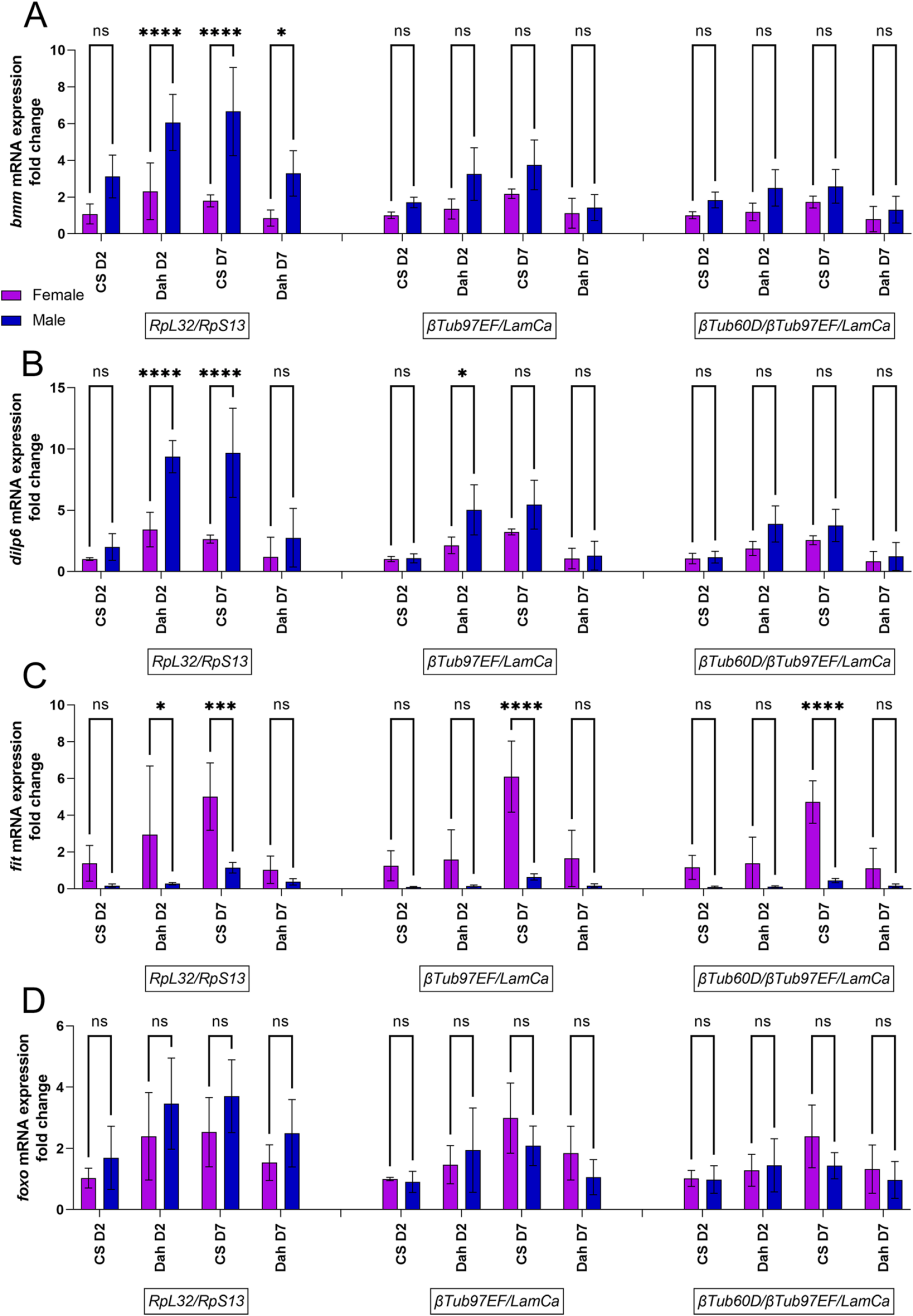
(**A**) Gene expression analysis for *Bmm* mRNA expression in body samples of Canton S10 (CS) or Dahomey (Dah) flies, 2 days (D2) or 7 days (D7) post eclosion in male and female flies. Gene expression was normalized using ribosomal genes as reference genes (RpL32/RpS13), *βTub97EF*/*LaminCa* or *βTub60D*/*βTub97EF*/*LaminCa*. Two-way ANOVA main effects as following: normalization; p < 0.0001; sex p < 0.0001 and their interaction; p = 0.003 with a Sidak’s multiple comparisons test comparing *Bmm* expression between males and females. ****p < 0.0001, *p < 0.05. (**B**) Gene expression analysis for *dIlp6* mRNA expression in body samples of Canton S10 (CS) or Dahomey (Dah) flies, 2 days (D2) or 7 days (D7) post eclosion in male and female flies. Gene expression was normalized using ribosomal genes as reference genes (*RpL32*/*RpS13*), *βTub97EF*/*LaminCa* or *βTub60D*/*βTub97EF*/*LaminCa*. Two-way ANOVA main effects as following: normalization; p < 0.0001; sex p < 0.0001 and their interaction; p < 0.0001 with a Sidak’s multiple comparisons test comparing *dIlp6* expression between males and females. ****p < 0.0001, *p < 0.05. (**C**) Gene expression analysis for *fit* mRNA expression in body samples of Canton S10 (CS) or Dahomey (Dah) flies, 2 days (D2) or 7 days (D7) post eclosion in male and female flies. Gene expression was normalized using ribosomal genes as reference genes (RpL32/RpS13), *βTub97EF*/*LaminCa* or *βTub60D/βTub97EF*/*LaminCa*. Two-way ANOVA main effects as following: normalization; p < 0.0001; sex p < 0.0001 and their interaction; p = 0.0008 with a Sidak’s multiple comparisons test comparing fit expression between males and females. ****p < 0.0001, ***p < 0.0005 *p < 0.05. (**D**) Gene expression analysis for *Foxo* mRNA expression in body samples of Canton S10 (CS) or Dahomey (Dah) flies, 2 days (D2) or 7 days (D7) post eclosion in male and female flies. Gene expression was normalized using ribosomal genes as reference genes (*RpL32*/*RpS13*), *βTub97EF*/*LaminCa* or *βTub60D/βTub97EF*/*LaminCa*. Two-way ANOVA main effects as following: normalization; ns, sex p < 0.0001 and their interaction; ns, with a Sidak’s multiple comparisons test comparing foxo expression between males and females.

**Figure 8:**
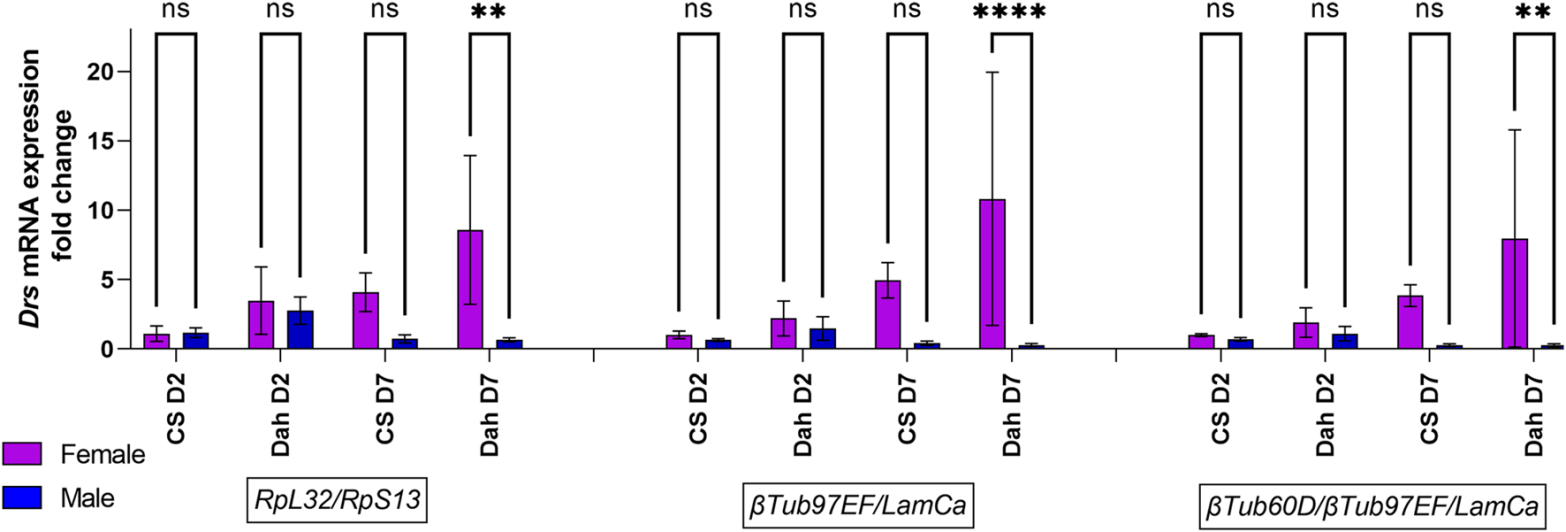
Gene expression analysis for *Drs* mRNA expression in body samples of Canton S10 (CS) or Dahomey (Dah) flies, 2 days (D2) or 7 days (D7) post eclosion in male and female flies. Gene expression was normalized using ribosomal genes as reference genes (*RpL32*/*RpS13*), *βTub97EF*/*LaminCa* or *βTub60D/βTub97EF*/*LaminCa*. Two-way ANOVA main effects as following: normalization; p = 0.025; sex p < 0.0001 and their interaction; p = 0.0021 with a Sidak’s multiple comparisons test comparing drs expression between males and females. ****p < 0.0001, **p < 0.005.

To validate this selection of reference genes, we used them to normalize the mRNA level (2^∆∆Cq^) of Insulin-like peptides, *dIlp2*, *dIlp3* and *dIlp5,* in head samples and *Brummer* (*Bmm)*, *female-specific independent of transformer (Fit)*, *Foxo, dIlp6* and *Drosomycin* (*Dros*) in body samples. Data are expressed as fold change compared to female CS10 at day 2 post-hatching. From Figs. 6, 7 and 8 it can be noted that the use of ribosomal subunit genes as reference genes induces a male bias or blunting of female biased expression. *dIlp2* expression in the head is not significantly different in males and females regardless of the chosen reference genes. However, the use of reference genes *LaminCa/αTub84B* or *LaminCa/αTub84B/eEF1α1* reveals a higher expression of *dIlp2* in female heads of Dahomey flies 7 days post eclosion, albeit not statistically significant. The same can be observed for *dIlp5*. The effect of reference genes is more evident for *dIlp3*, for which a female biased expression in head is seen in the FlyAtlas2 database. *dIlp3* is significantly higher in 7-day Dahomey female head samples compared to Dahomey 7-day male heads. These data suggest that *dIlp* expression can have sex bias, depending on the strain and age of the flies, and that reference genes chosen from ribosomal subunit genes might obscure the female bias. This phenomenon can also be observed in body samples analyzed for male-biased gene *Bmm*, where normalization using *RpS13/RpL32* leads to significantly higher expression of *Bmm* in males of Dahomey day 2 post eclosion and CS10 flies 7 days post eclosion. Previous studies noted a 1.8-fold higher expression of Brummer in male flies compared to female flies. This is indeed the fold change observed when normalized using reference genes *βTub97EF/LaminCa or βTub60D/βTub97EF/LaminCa*, while normalization with *RpS13/RpL32* leads to a 3-to-fourfold higher expression in males than females. A similar effect can be observed for *dIlp6* which is more highly expressed in males, but only with a factor 1.5, and not with a factor 2 to 3 which is obtained when normalized using *RpS13/RpL32. Foxo* expression appears to have a male bias when normalized using *RpS13/RpL32,* but a female bias when normalized using *βTub97EF/LaminCa or βTub60D/βTub97EF/LaminCa,* albeit not significant. Finally, we discovered a strong female bias in the gene *Drs* in flies 7 days post eclosion.

### Detection of sex-biased effects following a nutritional challenge

**Figure 9:**
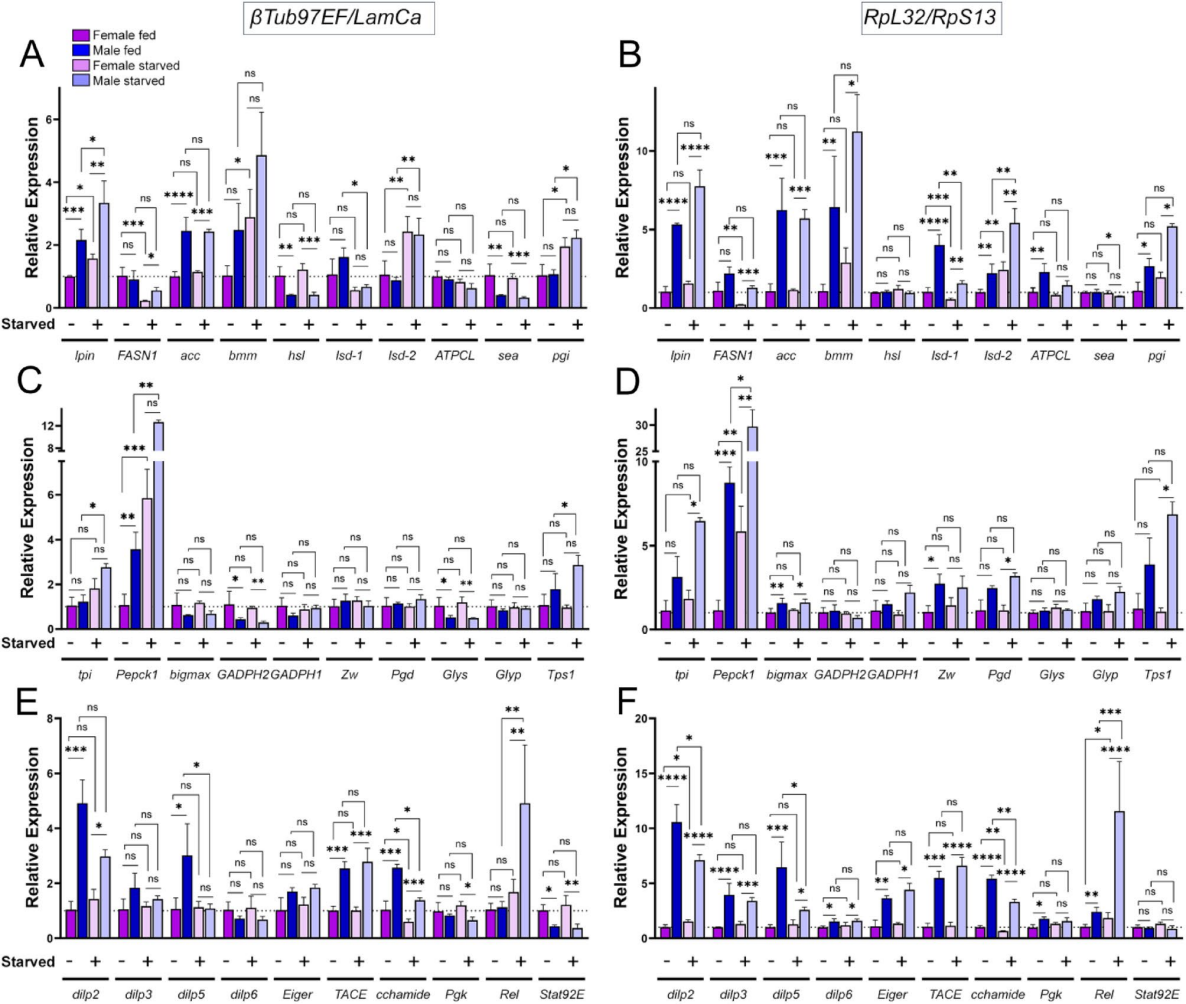
Gene expression analysis of metabolic genes according to sex and nutritional status in whole 7-day post eclosion Dahomey flies. Gene expression was normalized using structural genes *βTub97EF*/*LaminCa* (A, C, E) or ribosomal genes *RpL32*/*RpS13* (B, D, F) as reference genes. Two-way ANOVA with Sidak’s multiple comparisons test comparing gene expression between males and females, and between the same sex in both dietary conditions. ****p < 0.0001, ***p < 0.001, **p < 0.0, *p < 0.05 and *ns* not significant.

Metabolism is dynamically regulated by the nutritional state of the fly and this response can often be sex-biased^27^. We thus set out to check the validity of *βTub60D, βTub97EF, LaminCa, RpL32* and *RpS13* in a starvation context. We used a similar approach as for the incorporation of sex-bias effect in dCt values to determine if these reference genes were appropriate to compare gene expression in starved vs. non-starved flies. We calculated dCtStarved, which is the mean difference of Ct values of the reference gene between starved and non-starved animals. Multiplying this value by the stability values from the dCt method, and Normfinder, BestKeeper and GeNorm stability values, revealed that *LaminCa* and *βTub97EF* were the most appropriate for comparing starved versus non-starved animals. Only the GeNorm algorithm gave a higher preference for *RpL32* and *RpS13* (Supplemental Fig. 1A–H)*.* Consistently, *βTub60D* was found to be unstable upon starvation by all methods, *RpL32* and *RpS13*, which are sex-biased, were identified as being stable during starvation although most methods gave preference to *LaminCa* and *βTub97EF.* We next determined the effect of starvation on 30 different metabolic genes in both male and female Dahomey flies using *LaminCa* and *βTub97EF* as reference genes (Fig. 9A,C,E) or the combination of RpL32 and RpS13 (Fig. 9B,D,F). Comparison of these results demonstrates how the use of RpL32 and RpS13 as reference genes exaggerates the results for male gene expression. We found that several genes like *lpin, lsd-2* and *pgi* are dynamically regulated upon starvation in both sexes while others, like *FASN1, lsd-1* and *Rel,* show sex-biased effects upon starvation. Furthermore, other genes such as *hsl*, *sea*, *TACE* and *STAT92E* show sex-biased expression regardless of nutritional state.

The effect of normalization led to significant differences in interpretation like for *pgi,* which showed a male specific upregulation upon starvation when using ribosomal genes as reference genes, while normalizing it to *LaminCa* and *βTub97EF* showed that it is in fact upregulated in both sexes (Fig. 9A,B). In contrast, other genes like *hsl* and *GADPH2* showed female biased expression (Fig. 9A–D), no longer observed when they were normalized using *RpS13/RpL32.* Similar effects were observed in Canton S10 flies albeit with a higher level of variation between biological replicates (Supplemental Fig. 2A–F).

## Discussion

To accurately evaluate the expression of target genes in samples from different sexes, ages or strains it is crucial to select appropriate reference genes, displaying no bias towards a group of samples. In this study we have shown that in gene expression experiments on *Drosophila*, reference genes used for the normalization of target genes following qRT-PCR, display significant variation by sex, age and strain of the samples. We show that most of the commonly used reference genes (*18SrRNA, Actin42A, βTub56D, eEF1α1, Mnf, RpS20, RpL32* and *RpS13*) display a sex bias, with the exception of *αTub84B*. We included three other genes*. LaminCa*, *βTub97EF* and *Arp3,* also coding for structural components of the cell^32–34^, as new potential reference genes to a list of commonly used reference genes and evaluated their variation. Using the algorithms Normfinder, GeNorm, Bestkeeper and the deltaCt comparison we identified reference genes with the least variation across samples. The online tool RefFinder was not used in this study since it was reported to produce different outputs compared to the other software tools, due to the absence of information on the PCR efficiencies^16^. In head samples all algorithms proposed *LaminCa* as one of the three most stable reference genes, except for the results from Normfinder. For body samples *LaminCa* was one of the three most stable reference genes in all algorithm outputs, suggesting it is very stably expressed and potentially a suitable reference gene. *LaminCa* was not previously used as a reference gene in *Drosophila* samples. However, it has been used for normalization of rodent samples, and has been applied as potential biomarker in human tumor samples^35–37^. *βTub97EF* and *βTub60D* are members of the β-tubulin family which have been extensively used in insect and mammalian samples as reference genes^30,38^.

To take into account the sex-bias of reference genes, we used the method described by St-Pierre et al.^18^ and multiplied the difference in Ct-values with the stability values obtained from all 4 algorithms. This yielded *LaminCa*, *aTub84B* and *eEF1α1* for head samples and *LaminCa*, *βTub60D* and *βTub97EF* for body samples as the most stable genes in all calculations. These combinations of genes were used in a comparison to ribosomal protein-encoding genes as reference genes to evaluate the expression of genes associated with *Drosophila* metabolism in head and body samples. Ribosomal protein-encoding genes displayed a very strong female bias in body samples which may relate to the presence of functional ovaries and developing eggs^39^. These genes also displayed a female bias in head samples which may be partially explained by the presence of tissues such as head fat body. It was also previously reported that ribosomal protein-encoding genes display a difference in expression in *Drosophila* brain, with a female bias^40^.

We observed that the use of ribosomal protein-encoding genes as reference genes leads to artificially higher expression of target genes in males, blunting the female biased expression and increasing male biased expression to high fold changes, as compared to when the sex-bias-corrected reference genes were used. The use of the latter reference genes also yields expression results that are consistent with the differences found in the expression data (based on RNAseq) in the online database FlyAtlas2. This is obvious in body samples, where *Bmm* expression in males was more than three-fold increased when normalized using ribosomal protein-encoding genes (as described by Ref.^27^), but only 1.8-fold increased when normalized using the sex-bias corrected reference gene list. Also, for *dIlp6,* only a modest male bias, not statistically significant, was seen when using the sex-bias corrected reference genes. While there was no statistically significant difference in Foxo expression between males and females, it was slightly higher in males when the ribosomal protein-encoding genes were used for normalization. When using the sex-bias corrected reference gene list, the difference between males and females decreased, but a higher expression was observed in CS10 7 day old females compared to males. Few studies have evaluated the sex-difference in metabolic genes such as *dIlp6* or immune genes such as *Drosomycin*. We observed a very strong female bias in *Drs* expression in flies 7 days post eclosion, primarily in Dahomey flies. This was also reported by others for *Drosophila suzukii*^41^. Based on expression data available in FlyAtlas2, we propose that this female bias in *Drs* expression presumably stems from a very high expression of the gene in the spermatheca of mated females. In head samples, we evaluated the expression of *dIlp2, 3* and *5*. Insulin-like peptides play an essential role in the regulation of carbohydrate metabolism, lifespan and body size^42,43^. However, the sex-specific difference in the expression of these genes is only moderately studied and understood. Sex-bias has been reported for larval *dIlp3* expression with higher expression in males as compared to females, while no significant effect of sex was noted for *dIlp2* and *dIlp5*^44^. In a previous study, we also showed that *dIlp3* expression strongly increases in Dahomey males in response to sugar and fat added to the diet^29^. Remarkably, in this study, we observed a higher expression of *dIlp2, -3* and *-5* in 7-day-old Dahomey female heads, albeit only significant for *dIlp3*. The female bias we observed in Dahomey contrasts to what is reported in other *Drosophila* strains^45^.

Lastly, we evaluated the use of *βTub60D, βTub97EF* and *LaminCa,* identified in this work as sex stable, as reference genes in a starvation experiment. We found that *LaminCa* and *βTub97EF* are stable under starvation conditions while *βTub60D* showed considerable differences between fed and starved conditions, suggesting that the latter is regulated by nutrition. This further highlights the necessity of re-evaluating the use of reference genes when introducing a new variable. We next used *LaminCa* and *βTub97EF* as reference genes to measure the effect of 24 h starvation on metabolic gene expression in both male and female flies, and compared these results to those obtained when using *RpS13/RpL32* as reference genes. Using the ribosomal protein encoding genes to normalize the data did not dramatically alter the interpretation within the same sex when compared to performing the normalization with *LaminCa* and *βTub97EF,* but has a massive effect on the interpretation when comparing between sexes. In other insects, genes encoding for ribosomal genes have been identified as being stable during starvation^46,47^. In summary, this further illustrates the necessity of using appropriate reference genes as normalization performed using ribosomal protein genes resulted in significant male bias in the analysis.

Previous work has established that roughly 25% of transcripts are differentially expressed after starvation^48^. From our subset of 30 metabolism-related genes we found that 12 (40%) were up- or downregulated after starvation which reflects the dynamic response of metabolism on starvation^49^. Additionally, this response to starvation is sexually dimorphic, especially for genes related to gametogenesis but for metabolism as well^48^.

Overall, we conclude that the use of sex-bias corrected reference genes yields more accurate estimates of relative expression of genes in males and females and could thus contribute to furthering our understanding of the genetic basis of sex-associated differences in metabolism and this within and between strains.

## Materials and methods

### *D. melanogaster* lines and samples

Wild-type Canton S10 (kind gift of Dr. Ron Davis) and wild-type Dahomey (kind gift of Dr. Carlos Ribeiro) *Drosophila melanogaster* strains were reared at 25 °C on medium containing 5% w/v cornmeal, 2% w/v yeast, 0.5% w/v agar, 1.35% w/v dextrose, 3% v/v saccharose syrup, 0.75% v/v propionic acid, hydroxybenzoate and 1.125% ethanol. Freshly hatched flies were collected and kept on a fresh vial for 2 or 7 days at a 10:10 male—female ratio. Per sex, age, and strain, 3–4 replicates of 10 flies were collected in Eppendorf tubes, frozen in liquid nitrogen and immediately vortexed to separate heads from bodies (thorax-abdomen). Heads and bodies were collected in separate RNase-free Eppendorf tubes for RNA extraction. Samples were stored at − 80 °C until processing. For the starvation experiments 6-day-old Dahomey and Canton S10 flies were placed on starvation medium (1% agar) for 24 h. Whole flies were collected and processed.

### Use of FlyAtlas2 and FlyBase databases

An initial assessment of possible sex-biased expression of commonly used reference genes was done by mining the FlyAtlas2 database^50^ (http://flyatlas.gla.ac.uk/FlyAtlas2/index.html, released August 2, 2022). FlyBase was also used to select genes annotated as belonging to “structural components of the cell or nucleus” (www.flybase.org, FB2022_04, released August 8, 2022)^31^.

### Primer design and primer efficiency

Primers were designed with the NCBI Primer-BLAST tool (https://www.ncbi.nlm.nih.gov/tools/primer-blast/). Primer sequences and primer efficiencies are listed in Table 1. Primer efficiencies were determined for each primer pair using cDNA samples derived from pools of 10 heads or 10 bodies (thorax + abdomen) from female Dahomey flies. RNA extraction and cDNA synthesis were performed as described in the next subsection of this Material and Methods. cDNA samples were diluted 1/5, 1/10, 1/50, 1/100, 1/1000, 1/10 000 to determine the dynamic range of the standard curve. Quantitative RT-PCR was performed on the undiluted and diluted samples for all reference and experimental genes. Ct-values were plotted, and the linear relationship was determined. Primer efficiency was calculated using the following formula: $Efficiency \left(\%\right)={(10}^{-\frac{1}{slope}}-1) x 100$.

### RNA extraction, cDNA synthesis and qPCR-PCR

Total RNA was isolated using phenol–chloroform extraction. Briefly, heads or bodies were homogenized in 1 ml Tri-Sure^®^ (GC-Biotech, Waddinxveen, The Netherlands) with plastic pestles. Samples were incubated for 5 min at room temperature. 200 µl RNase-free chloroform was added to the tube, which were then shaken vigorously by hand. The samples were incubated for 3 min at room temperature followed by centrifugation at 10000 *g* or 15 min at 4 °C. The upper aqueous phase was isolated and transferred to a new RNase free Eppendorf containing 500 µl isopropanol. Samples were gently inverted to mix the aqueous phase with isopropanol, followed by 10 min centrifugation at 12000 *g* at 4 °C. The supernatant was removed, the pellet washed with 1 ml of 75% ethanol by centrifugation at 7500 *g* for 5 min at 4 °C. Supernatant was removed completely and the pellet was air-dried for 5 min at room temperature and resuspended in 22 µl RNase free water for heads and 102 µl for bodies. RNA was stored at − 80 °C until further processing. RNA concentrations were measured with a Nanodrop ND-1000 spectrophotometer (Thermo Fisher Scientific, Wilmington, U.S.A.). cDNA was produced using SensiFAST™ cDNA synthesis kit (GC Biotech, Waddinxveen, The Netherlands) in 20 µl total volume using 1 µg of total RNA, following the manufacturer’s protocol. cDNA was diluted tenfold in nuclease free water to a final concentration of 100 ng/µl and was stored at − 20 °C until used. qRT-PCR reactions were performed on a ViiA 7 Applied Biosystems Real-Time PCR system (Thermo Fisher Scientific, Wilmington, U.S.A.) and on a Quantstudio 6 Pro (Thermo Fisher Scientific) using SYBR Green (FastGene 2 × IC Green mix—low ROX, Nippon Genetics, Düren, Germany) with 200 ng cDNA template and 100 nM of each primer, in 384-well optical plates.

### Data analysis

Expression levels were determined as the number of cycles (cycle threshold, Ct-value) needed for the amplification to reach a fixed threshold in the exponential phase of the PCR reaction^51^. The threshold was set at 0.04 for all genes, and the corresponding Ct-values were transformed into quantities via the standard curve using PCR efficiencies according to Ref.^13^. To determine the expression stability of the selected reference genes, we used the following methods: Delta Ct method^12^ geNorm^13^, NormFinder^15^, BestKeeper^14^. For the analyses with the geNorm and NormFinder procedures, the individual Ct values were transformed to relative quantities by calculating 2^(Ct–Ctminimum)^. For calculations using the BestKeeper and delta Ct method, the untransformed Ct-values were used. Calculations were done in Excel (Delta Ct, Normfinder, Bestkeeper) or qBase + (geNorm).

Following the identification of the best reference gene according to the software used, the gene expression ratio was determined according to the PfaffI method^14^ to assess the expression of metabolism-associated genes, i.e. *dIlp2, 3* and* 5* in head samples, *Bmm, Foxo, fit* and *dIlp6* in body samples, and the immune-associated gene *Drs* in body samples. The relative quantity of the gene of interest was measured and normalized relatively to that of the validated reference gene using the following formula:

$$ratio=\frac{{e}^{-\mathrm{Ct }\left(\mathrm{mean\, CT\, controlGOI}-\mathrm{mean\, ct\, sampleGOI}\right)}}{{e}^{-\mathrm{Ct }\left(\mathrm{mean \,CT \,Refcontrol}-\mathrm{mean\, ct\, sampleREF}\right)}}$$

### Statistical analysis

Graphpad Prism 9.2.0 was used for the statistical analysis of the data with two-way ANOVA with Sidak’s multiple comparisons test.

### Supplementary Information

Supplementary Figure 1.

Supplementary Figure 2.

## Data Availability

All data are included in the manuscript.
