# Supplementary material for: Reference genes to study the sex-biased expression of genes regulating Drosophila metabolism
Source: Sci Rep. 2024 Apr 25;14:9518. doi: 10.1038/s41598-024-58863-5 (PMC11045863; doi:10.1038/s41598-024-58863-5)
Supplement: Supplementary file 1 — Supplementary Figure 1. [file 41598_2024_58863_MOESM1_ESM.pdf]

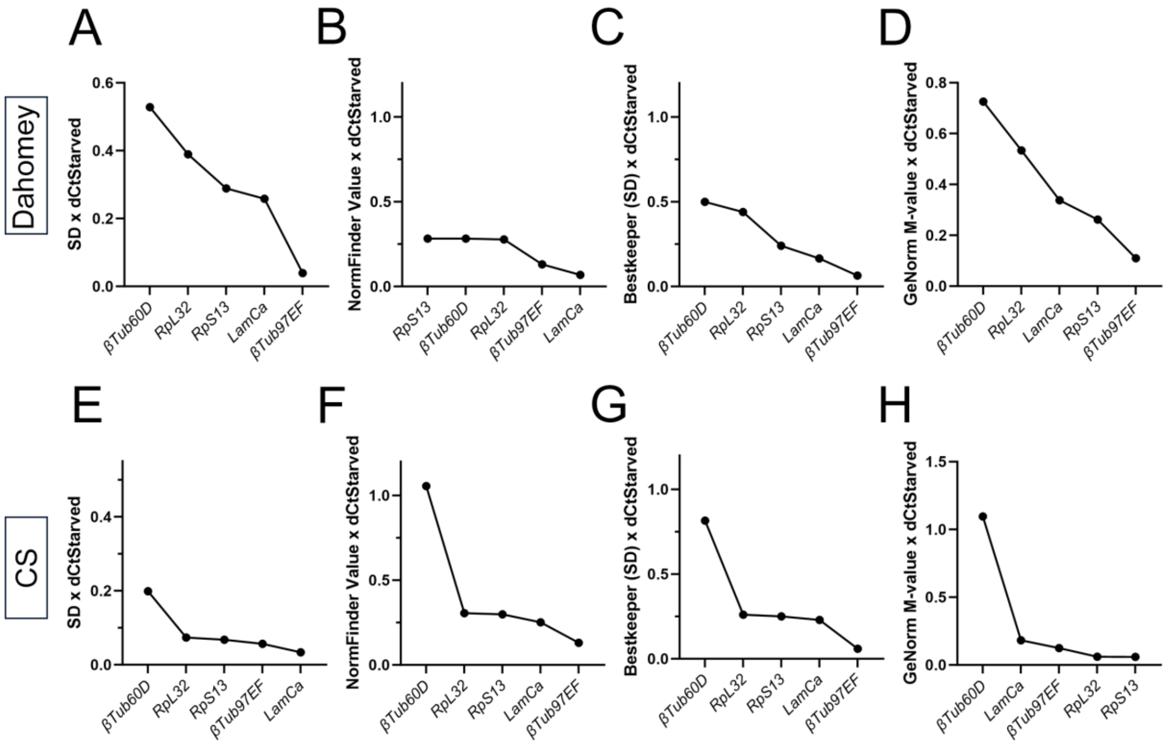

Supplemental Figure 1

Graphs depicting the calculation of most stable reference genes according to delta-Ct method (A and C) and normfinder (B and D), multiplied with the absolute difference in mean CT value of the reference genes in fed versus starved flies (deltaCtStarved) in Canton S10 and Dahomey flies.
