# Supplementary material for: Reference genes to study the sex-biased expression of genes regulating Drosophila metabolism
Source: Sci Rep. 2024 Apr 25;14:9518. doi: 10.1038/s41598-024-58863-5 (PMC11045863; doi:10.1038/s41598-024-58863-5)
Supplement: Supplementary file 2 — Supplementary Figure 2. [file 41598_2024_58863_MOESM2_ESM.pdf]

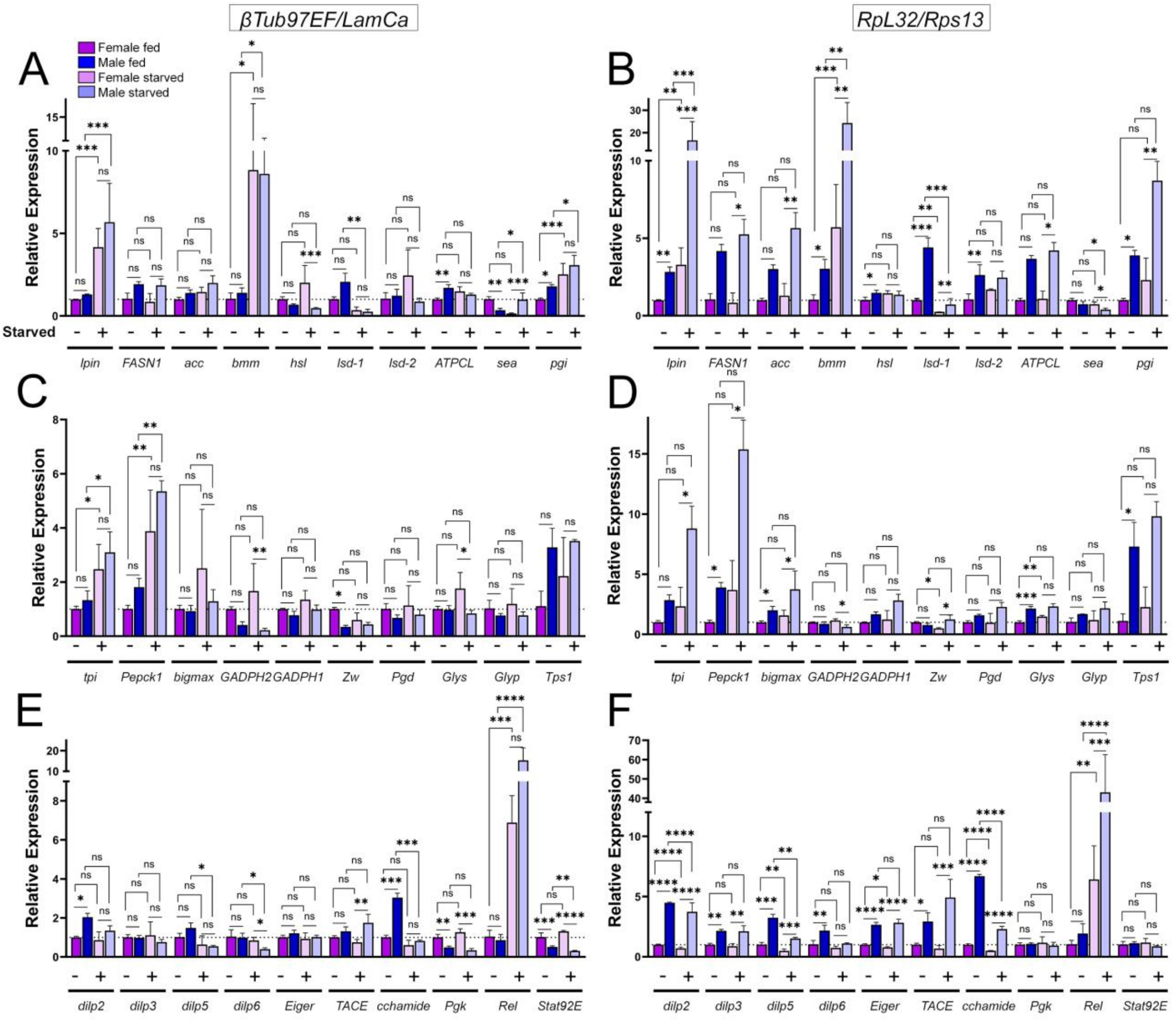

Supplemental Figure 2

Gene expression analysis of metabolic genes according to sex and nutritional status in whole 7-day post eclosion Cantons S10 flies. Gene expression was normalized using structural genes betaTub97EF/LaminCa (A, C, E) or ribosomal genes RpL32/RpS13 (B, D, F) as reference genes. Two-way ANOVA with Sidak's multiple comparisons test comparing gene expression between males and females, and between the same sex in both dietary conditions. **** p<0.0001, ***< 0.001, ** p<0.0, * p<0.05 and ns not significant.
